# Synchronous Presentation of a Cervical Spinal Schwannoma and Primary Progressive Multiple Sclerosis in a 65-year-old Man

**DOI:** 10.7759/cureus.4176

**Published:** 2019-03-04

**Authors:** Avital Perry, Pierce Peters, Christopher S Graffeo, Lucas P Carlstrom, William E Krauss

**Affiliations:** 1 Neurologic Surgery, Mayo Clinic, Rochester, USA; 2 Neurological Surgery, Mayo Clinic, Rochester, USA

**Keywords:** spinal schwannoma, multiple sclerosis, differential diagnosis, schwannomatosis

## Abstract

Schwannomas are common benign neoplasms of the myelinating cells surrounding peripheral nerve axons. Though uncommon, lesions arising in the cervical spine may produce radicular pain and myelopathic weakness. Multiple sclerosis (MS) is a common autoimmune disorder with the capacity to mimic nearly any neurologic disease, including spinal cord neoplasms. We report the third case of synchronously presenting primary progressive MS and spinal schwannoma.

A 65-year-old man presented with six months of progressive weakness and pain of the right shoulder, forearm, and hand. MRI demonstrated a contrast-enhancing transforaminal lesion at C7, most consistent with a benign nerve sheath tumor. Additional history disclosed several years of worsening fatigue, accompanied by bilateral weakness and lancinating leg pain. MRI of the neuraxis demonstrated abnormalities consistent with chronic demyelinating disease intracranially and within the spinal cord; cerebrospinal fluid (CSF) analysis revealed nine oligoclonal bands and an elevated IgG index, resulting in the diagnosis of MS. Given the symptomatic C7 lesion, the patient subsequently underwent right C6-C7 facetectomy, gross total resection of the tumor, and C6-T1 posterior instrumented fusion. Postoperatively, the patient rapidly recovered normal right upper extremity function, and pathology confirmed benign schwannoma.

Synchronously presenting co-morbid neurologic diagnoses are exceedingly rare. Nonetheless, the high incidence and protean nature of MS make it particularly susceptible to such confounding clinical cases. Correspondingly, MS should be considered when neurologic abnormalities are not compatible with a focal radiographic lesion, and the present report emphasizes the value of a good history and exam in unraveling similarly challenging cases.

## Introduction

Spinal schwannoma is the most common intra-dural extra-axial neoplasm, comprising nearly one-third of tumors found within that anatomic space, with an incidence of 0.3-0.5 per 100,000, and a peak distribution during the fifth decade of life [1-3]. Multiple sclerosis (MS) is a highly prevalent demyelinating disease, occurring at an incidence of two per 100,000, with a mean age of 30 and a 2.3:1 female predominance [4-5]. Considered one of the “great imitators,” MS manifests with a wide swath of confounding symptoms, and has been mistaken for nearly every other common neurologic diagnosis, including spinal tumors [6-7].

Although far from universally applicable, Dr. William Osler’s repurposing of Occam’s Razor-that a single, unifying diagnosis should be sought to explain all of a given patient’s symptoms-remains salient, and in many cases provides a helpful tool for scrutinizing the differential and eliminating diagnoses that are simultaneous unlikely and supported [8]. Notwithstanding, as Dr. John Hickam proposed in his counterpoint to Osler’s Rule, Hickam’s Dictum, there are numerous circumstances, both pedestrian and rarified, that defy simplicity, and that practical, clinical evidence should universally trump theoretical postulates, allowing the patient to have “as many diagnoses as he damn well pleases” [9]. In the spirit of this dialogue, we present a rare case of synchronously presenting spinal schwannoma and multiple sclerosis-only the third such patient-reported, and an important illustration of the principles of attentive diagnosis with a detailed history and physical exam, resistance of radiographic search satisfaction, and openness to the unexpected, particularly where the clinical evidence flies in the face of Occam’s Razor.

## Case presentation

**Figure 1:**
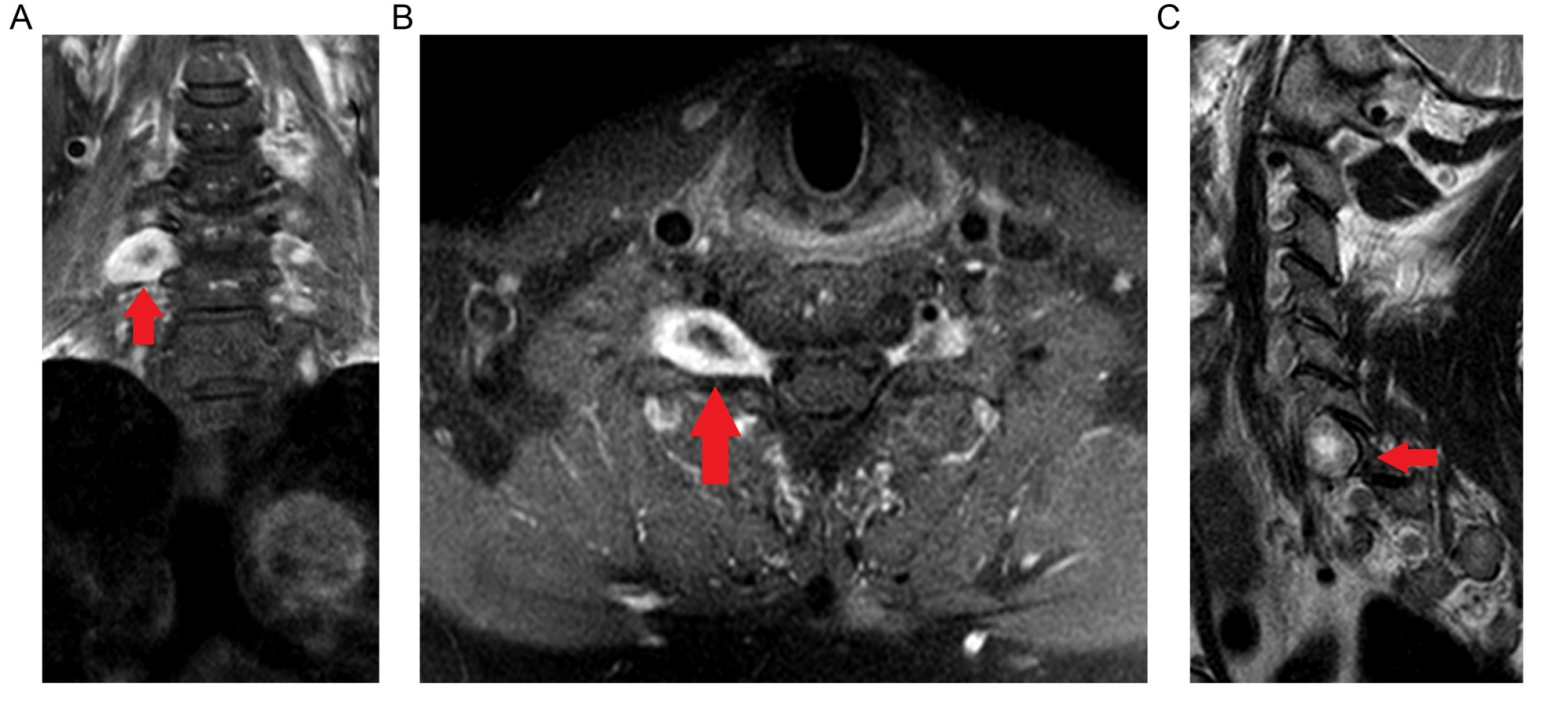
Gadolinium-enhanced T1-weighted coronal (A), axial (B), and T2-weighted sagittal (C) MRI signal highlighting a centrally cystic/necrotic nerve sheath tumor at the level of C7 (arrows).

A 65-year-old man presented with six months of progressive weakness and pain of the right shoulder, forearm, and hand. He was evaluated by his local neurologist and neurosurgeon, and an MRI was acquired and demonstrated findings consistent with a C7 nerve sheath tumor (Figure 1). Electromyogram (EMG) confirmed right C7 radiculopathy, and the patient was referred to our institution for neurosurgical treatment. Although congruent findings were noted on MRI and EMG, additional history was obtained that revealed highly concerning findings inconsistent with a cervical nerve root schwannoma. The patient had several years of progressively worsening fatigue with activity, bilateral leg weakness, and shooting pain, as well as bladder urgency without incontinence. Physical examination revealed right C7 weakness, marked gait unsteadiness, and positive Babinski sign on the left.

**Figure 2:**
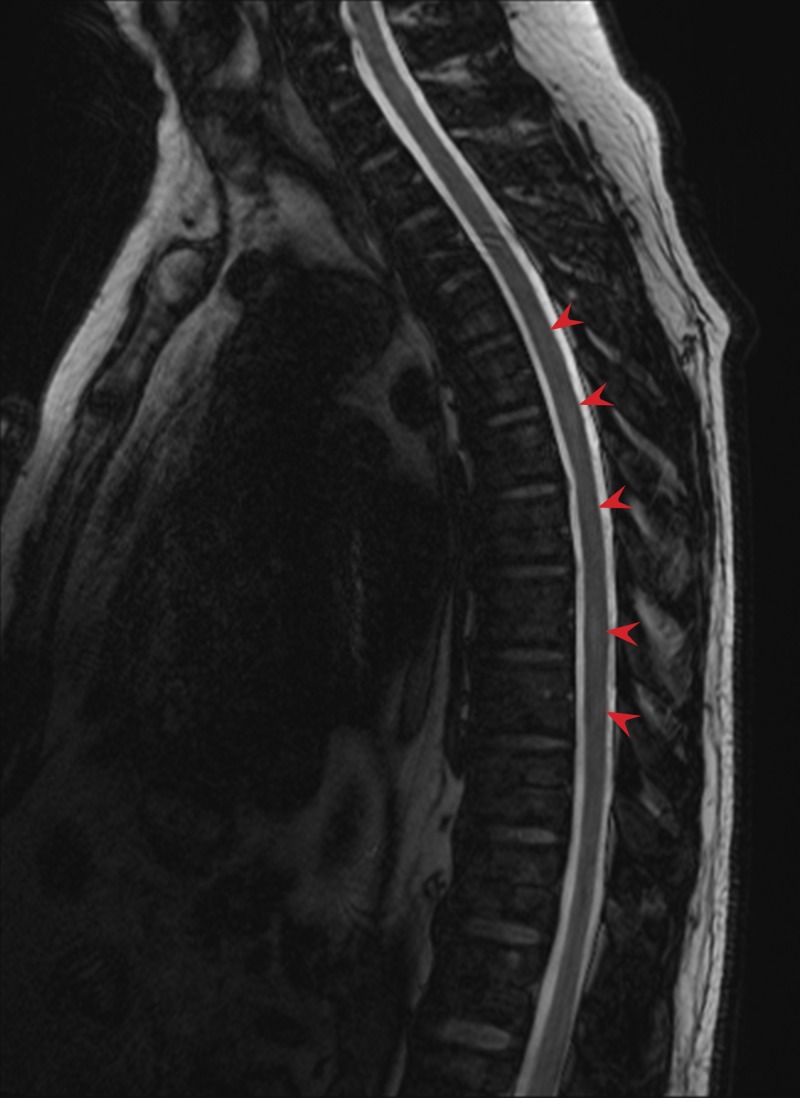
Indeterminate signal abnormality at multiple levels (arrows) of the thoracic and lumbar spine on T2-weighted, Dixon water-fat opposed MRI.

**Figure 3:**
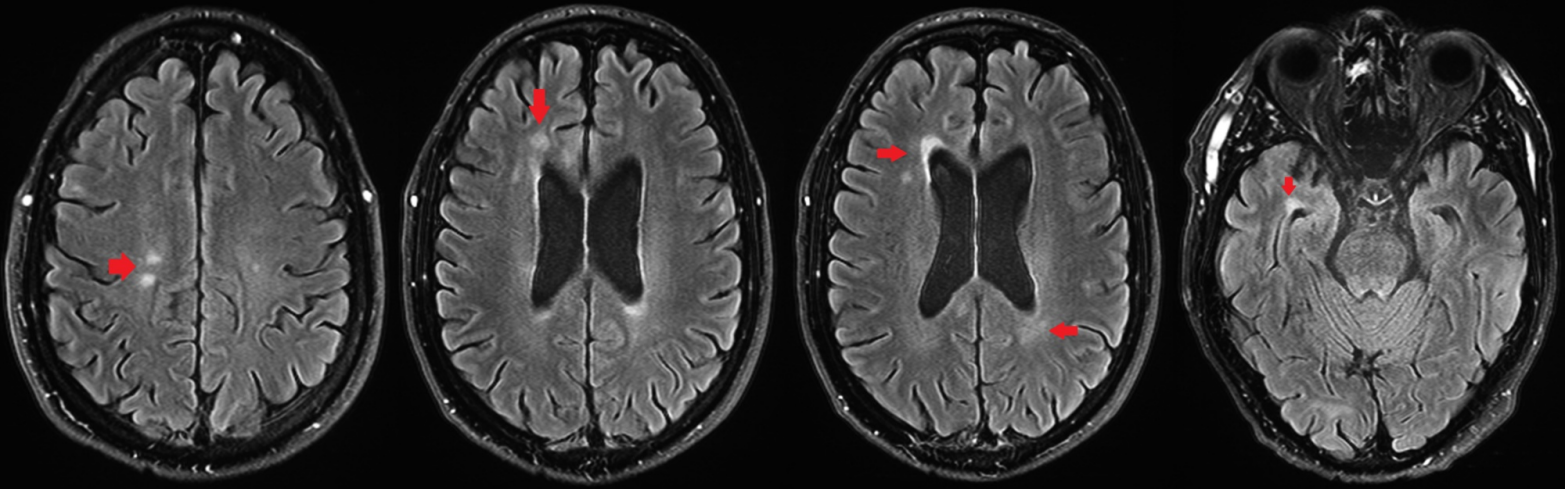
Axial fluid-attenuated inversion recovery (FLAIR) T2-weighted, fat-suppressed brain MRI demonstrating multiple foci of juxta-ventricular signal hyperintensity (arrows).

These new findings prompted MRI of the total neuraxis, which reproduced the right C7 mass, while also identifying subtle, intrinsic T2 signal abnormalities of the thoracic spinal cord (Figure 2), and multiple periventricular T2 signal abnormalities (Figure 3), collectively raising significant concern for chronic demyelinating disease. Cerebrospinal fluid (CSF) analysis identified had nine oligoclonal bands with an elevated IgG index, and the diagnosis of primary progressive MS was made. Of note, the patient was assessed for benign prostatic hyperplasia, and it was thought unlikely to be responsible for his presenting urinary symptoms.

Although the diagnosis of MS provided a satisfactory explanation for the patient’s otherwise confounding symptoms, he remained with a demonstrably symptomatic cervical schwannoma, now complicated by the possible need for future immunosuppression. Correspondingly, the tumor was approached via a right posterior C6-C7 facetectomy; a gross total resection of the tumor was achieved; and a C6-T1 posterior instrumented fusion was completed, due to concern for possible destabilization in the setting of total facetectomy at a spinal level proximal to the cervicothoracic junction. Pathology confirmed a schwannoma. By post-operative day one, his right upper extremity weakness and radiculopathy were dramatically improved and had resolved by first neurosurgical follow-up at six weeks. The neurology service determined that the extent of his MS disease burden was such that initial management could be expectant; correspondingly, immunosuppression was not recommended and his other symptoms remained stable as of the last neurosurgical follow-up at one year.

## Discussion

Although both spinal schwannomas and MS are relatively common diagnoses, their synchronous presentation is exceedingly rare, with ours marking only the third reported case. Moreover, our review of the literature did not find report of other demyelinating conditions such as neuromyelitis optica or transverse myelitis presenting synchronously with schwannoma, or MS coexisting with an extraspinal schwannoma. Schwannomas are benign neoplasms arising from the myelinating Schwann cell found throughout the peripheral nervous system, with a strong predilection for the cerebellopontine angle and the spinal nerve roots. When arising from spinal nerves, radiculopathy is the typical presentation, beginning with dermatomal pain, which may evolve into progressive focal motor weakness as the tumor grows and mass effect increases [10]. MRI findings include a characteristically well-circumscribed, contrast-enhancing mass, with T2 hyperintensity, and frequent involvement of the neural foramen at the level of disease [2]. More than 95% of spinal schwannomas occur sporadically; however, several distinct syndromic presentations are frequently observed [11]. Schwannomatosis is a recently described syndrome defined by multiple schwannomas without presence of a vestibular tumor, and although the overall incidence is rare (0.58 cases per one million), spinal involvement is highly prevalent among patients, with three in four harboring at least one spinal nerve root tumor [12]. In parallel, patients with the well-described neurocutaneous phakomatosis neurofibromatosis type two (NF2) very frequently develop spinal schwannomas, accounting for 1-2% of all such tumors [1, 11]. For both the sporadic and the syndromic diagnoses, the most common initial symptoms are localized pain, paresthesia/numbness, and motor weakness. Standard-of-care is almost universally surgical resection, although minimally symptomatic lesions can be safely observed, and radiotherapy may be considered for patients who are poor surgical candidates.

MS is a truly protean disease, with common presenting symptoms spanning the breadth of neurologic dysfunction, including visual loss, diplopia, sensory disturbance, focal motor weakness, bowel/bladder dysfunction, ataxia, and other cranial neuropathies [13]. Correspondingly, effectively every conceivable neurologic symptom has been described in MS, including the combination of dermatomal pain, motor weakness, and hyporeflexia that are characteristic of a spinal schwannoma [14]. Given the high degree of variability in clinical, radiographic, and laboratory findings associated with MS, diagnosis is made using the McDonald Criteria, which require dissemination of lesions in time and space [15]. In the modern era, MRI has become sufficiently advanced to date multiple lesions as major timepoints-for example, contrast enhancement is visualized at a different point in the natural history of an MS plaque than non-enhancing T2 hyperintensity, and so simultaneous enhancing and T2-bright lesions are radiographically “separated in time.” However, as with the disease’s clinical manifestations, the imaging findings are not universally pathognomonic, and MS lesions have been described with radiographic appearances ranging from a spinal cord or nerve root tumor to CNS lymphoma, glioblastoma, metabolic derangements, and a host of other neurologic disorders [16-17]. Consequently, MS is frequently upheld as one of the great pathologic imitators-although it is far more commonplace to find MS in lieu of another diagnosis it was imitating than in addition to it [18].

With these disease features in mind, we turn to the two previously reported cases of concomitant presenting spinal schwannoma and MS. In the index case, Salvi et al. described a 32-year-old man with progressive pain and weakness of the left lower limb, which eventually evolved to encompass the right lower extremity, with simultaneous urinary urgency [19]. MRI of the brain demonstrated multiple white matter lesions concerning for demyelinating disease, while T-spine imaging identified a vividly contrast-enhancing intra-dural extramedullary lesion at T7, which was subsequently resected and confirmed as a spinal schwannoma. Subsequently, Etus et al. described a 46-year-old woman with new right lower extremity paresthesias and urinary incontinence, as well as a two-year history of MS [20]. Physical examination revealed sensory disturbance in a T9 sensory distribution with bilateral deep tendon hyperreflexia. When these symptoms did not improve with corticosteroid therapy, MRI of the spine was performed, and an intra-dural extramedullary contrast-enhancing mass was identified on the right-sided T10 nerve root, which was surgically resected and diagnosed as a schwannoma. Although the diagnosis of MS was already established in this case, it provides another important illustration of the key clinical principle: that the simplest answer should not universally be assumed as correct, and, from a practical perspective, repeat MR imaging is requisite before a new symptom in a patient with MS can definitively be considered an expression of that primary diagnosis. 

## Conclusions

In many clinical circumstances, Osler’s Rule proves a still-relevant diagnostic principle-particularly with respect to neurologic diagnoses such as MS, which have the capacity to elicit simultaneous symptoms across a range of anatomically disparate domains, from sensation to locomotion, vision to micturition. Notwithstanding, although a single pathology is undoubtedly the “simpler” and more gratifying explanation to a diagnostic dilemma, clinicians are obliged to weigh all the evidence and to seek out a solution or set of solutions that incorporates all the pertinent history, exam, and imaging findings. As such, our report is not only the third case of synchronously presenting MS and spinal schwannoma, but a compelling reminder of the capacity for atypical cases to confound, and one which emphasizes the importance of bringing fresh eyes and an unbiased perspective to every neurosurgical referral.
